# Seasonal Dynamics of Aphid Flights and Cotton Leafroll Dwarf Virus Spread in Alabama

**DOI:** 10.3390/insects14070604

**Published:** 2023-07-04

**Authors:** Jessica B. Mahas, Charles Ray, Adam Kesheimer, Kassie Conner, Alana L. Jacobson

**Affiliations:** 1 Department of Entomology and Plant Pathology, Auburn University, 301 Funchess Hall, Auburn, AL 36849, USA; jba0022@auburn.edu (J.B.M.); chr138@msstate.edu (C.R.); ajk0055@auburn.edu (A.K.); 2 Alabama Cooperative Extension, 961 S. Donahue Dr., Auburn, AL 36849, USA; connekn@auburn.edu

**Keywords:** aphids, virus epidemiology, plant viruses, cotton, population dynamics

## Abstract

**Simple Summary:**

Cotton leafroll dwarf virus (CLRDV) can cause yield loss of cotton in susceptible plants. The only known aphid vector in the U.S. is *Aphis gossypii*, which regularly infests cotton, but knowledge so far is limited to the cotton growing season. Seven other species are also known to feed on cotton, but little is known about their seasonal dynamics. Two species are known vectors of CLRDV in chickpea in India. Improving our understanding of the viral spread and seasonal dynamics of potential vectors is key to developing management strategies for plant pathogens. In this study, we monitored seasonal population dynamics of these eight species, in three locations and two different habitats in Alabama, with the use of pan traps, year-round, for two consecutive years. To coordinate this with viral presence, CLRDV spread was monitored at the same sites with the use of cotton sentinel plants. Plants (while conditions allowed) and traps were collected and replaced weekly. Our findings provided baseline information on when these eight species are dispersing in the landscape and when CLRDV spread is occurring in cotton. While *A. gossypii* is likely the primary vector, early season spread of CLRDV suggests additional aphid vectors are possible.

**Abstract:**

Cotton leafroll dwarf virus (CLRDV) is an introduced Polerovirus (Family: Solemoviridae) of cotton, *Gossypium hirsutum* L., in the U.S. The only vector known to transmit this virus to cotton is the cotton aphid, *Aphis gossypii* Glover; however, there are seven other species of aphids (Hemiptera: *Aphididae*) reported to colonize cotton in the southeastern U.S.: *Protaphis middletonii* (Thomas), *Rhopalosiphum rufiabdominale* (Sasaki), *Aphis craccivora* Koch, *Macrosiphum euphorbiae* Thomas, *Myzus persicae* (Sulzer), *Smythurodes betae* Westwood, and *Aphis fabae* Scopoli. Little to no information is available on annual population dynamics of these species in the southeastern U.S. The timing of CLRDV spread to cotton plantings is also unknown. The objective of this study was to monitor the population dynamics of eight cotton-feeding aphid species concurrent with the spread of CLRDV at three different locations in Alabama. Aphids were monitored weekly for two years with yellow pan traps, and sentinel plants were deployed weekly to monitor CLRDV spread throughout the cotton-growing season. During the two years, most CLRDV spread at all locations occurred when *A. gossypii* was actively dispersing in the field. Early season spread at sites in south and central Alabama, when *A. gossypii* was not abundant, suggests additional aphid vectors are possible.

## 1. Introduction

Cotton, *Gossypium hirsutum* L., fiber production generated over $7 billion in the U.S. economy in 2021 [1]. An introduced Polerovirus (Family: Solemoviridae), cotton leafroll dwarf virus (CLRDV), has been identified in the U.S. cotton belt [1,2,3,4,5,6,7,8,9] and has the potential to cause yield reductions [3,10,11]. Viruses in this family are transmitted by aphids (family: Aphididae) in a persistent and circulative manner and are often transmitted for the lifespan of the vectors [12]. Three aphid species have been shown to transmit CLRDV, but only *Aphis gossypii* Glover annually colonizes cotton and is known to transmit the virus to cotton [12,13,14]. Besides *A. gossypii*, seven other aphid species have been occasionally observed on cotton in the U.S. [15], and evidence suggests that additional CLRDV vectors may be involved [16]. However, limited information exists regarding population dynamics of aphid species in cotton and virus spread.

Final CLRDV virus incidence has been shown to be as high as 80–100% in some areas in Alabama and Georgia using RT-PCR [12,17]. This suggests high numbers of viruliferous *A. gossypii*, multiple vectors, or multiple transmission events are present during the growing season. The symptomatology of the disease caused by CLRDV and yield losses are variable [3,11,16], and asymptomatic infections occur [11]. RT-PCR is required to determine the infection status of tested plants, and infections are not detected until 30–60 days after inoculation [16,18]. Twenty-three weed species from sixteen different families have been identified as alternate hosts for CLRDV in the U.S. These findings show CLRDV can infect common weeds found in cotton agroecosystems and is established in the landscape [19]. *Aphis gossypii* has been shown to transmit CLRDV between cotton and alternate hosts hibiscus (*Hibiscus acetosella* Welw. Ex Hiern.), okra (*Abelmoschus esculentus* L. cv. “Clemson Spineless 80”), amaranth (*Ameranthus palmeri* S. Watson), and prickly sida (*Sida spinosa* L.), in addition to these plants supporting aphid colony development [17]. In addition, *Aphis gossypii* apterous and alate morphs can acquire the virus in 30 min and 24 h, inoculate in 45 and 15 min, and retain CLRDV for 15 and 23 d, respectively [14,20]. Quick acquisition and transmission times, coupled with long retention periods, suggests why insecticide management of *A. gossypii* was shown to be ineffective at reducing final virus incidence [16]. Transient vectors that feed on but do not colonize a crop can also contribute significantly to the virus’ spread [21,22]. Data suggests transient vector species may be involved in CLRDV transmission, as viral spread was observed in the early and late season in Alabama when *A. gossypii* abundance in the field was low, and no other species were observed on cotton [16]. A better understanding of the timing of CLRDV spread to cotton and the number of vectors in the landscape is needed to advance knowledge on the epidemiology of CLRDV and guide development of effective management strategies.

Of the eight aphid species reported to colonize cotton in the U.S. [15,23], *A. gossypii* is the only reported vector of CLRDV [13]. This species annually colonizes cotton [24] and feeds on hosts from at least 25 plant families [23]. While reproduction on cotton is usually parthenogenic, sexual reproduction has been reported on alternate hosts, e.g., *Hibiscus*, in cooler environments [25]. The other seven species are rarely observed on cotton, are not considered to be economically important, and have not been studied in cotton agroecosystems. *Myzus persicae* (Sulzer) and *Aphis craccivora* Koch have been shown to transmit CLRDV to chickpea (*Cicer arietinum* L.) in India [26,27]. Single populations of *M. persicae* and *A. craccivora* tested for vector competence in the U.S. did not acquire CLRDV from, or transmit it to, cotton [20]. Vector competence to transmit CLRDV is unknown for *Aphis fabae* Scopoli, *Symthurodes betae* Westwood, *Macrosiphum euphorbiae* Thomas, *Protaphis middletonii* (Thomas), and *Rhopalosiphum rufiabdominale* (Sasaki). All are known to transmit at least one plant virus, and *P. middletonii* (as *A. armoraciae* Cowen) is reported to transmit several different viruses [23]. Knowledge of these species’ seasonal dynamics in cotton agroecosystems coupled with information about the timing of the virus’ spread is needed to identify possible transient vectors and to understand the seasonality of CLRDV spread in the landscape. Aphid monitoring using yellow pan traps, coupled with using healthy cotton seedlings as sentinel plants, is an effective way to monitor the seasonal dynamics of aphids and the timing of CLRDV spread [16], but this has previously been conducted for one year during the cotton growing season [16]. 

We monitored the seasonal dynamics of eight cotton-feeding aphids concurrent with CLRDV spread in three distinct cotton-producing regions of Alabama. We developed information regarding which aphids are dispersing, when they are dispersing into cotton plots, and when virus spread to cotton is occurring and associated aphid dispersal and virus spread. This is a descriptive biology study aiming to determine whether viral spread is occurring, and, when it does, what species are active during this time. 

## 2. Materials and Methods

Aphid dispersal and CLRDV spread were monitored in each of three distinct regions in Alabama that vary in geography, ecoregion, and climate [28] that can influence species presence, abundance, and population dynamics. The ‘South AL’ location was at the Brewton Agricultural Research Unit (BARU) in Brewton, AL. The ‘Central AL’ location was at the E.V. Smith Field Crops Research Center (EVS) in Shorter, AL. The ‘North AL’ location was at the Tennessee Valley Regional Research and Extension Center (TVREC) in Belle Mina, AL. Both the south and central locations are classified in the Environmental Protection Agency (EPA) Southeastern plain regions and USDA Hardiness Zone 8a [28,29]. This region can have poor soil drainage, with heavy clay that can be sticky when wet but cracks when dry. The northern location is located within an EPA interior plateau region, in USDA Hardiness Zone 7b [29]. This region is rich in farmland and has an abundance of shale and limestone [28]. Compared to South and Central AL, the location in North AL has average cooler minimum and maximum temperatures and the differences in soil types cater to distinct fauna. At each location, aphids and CLRDV were monitored around the perimeter of one cotton field and one non-crop area (pasture or weedy field). Including a non-crop area allowed us to ensure we were capturing aphids that were dispersing in the landscape during the cotton growing season, whereas aphids and CLRDV detected solely around the cotton field may have originated from the cotton field itself. This was justified because CLRDV is established in the landscape in various weeds around agricultural fields [17]. Cotton was managed according to standard local recommendations [30].

Aphids were monitored weekly using yellow pan traps (21 cm diameter × 7.5 cm height) from March 2020 to March 2022 according to methods of Mahas et al. [16]. Traps were filled with 50% propylene glycol and a drop of liquid dish soap each week after aphids captured the previous week were collected. Traps were collected every seven days and the contents were stored in 70% ethanol for transport and storage. Year one was the time period from mid-March 2020 until mid-March 2021, and year two was the remainder of the time from mid-March 2021 through mid-March 2022. Four yellow pan traps were placed around a non-crop and crop area at each location, for a total of eight pan traps per location. Traps were out year-round in the non-crop habitat, but were removed from the cotton field for harvest and were not reinstalled into their field positions until the following spring; the cotton growing periods for each location are listed in Table 1. 

Four healthy two to three true-leaf cotton seedlings (Var. DP1646, DeltaPine^®^, Dekalb Genetics Corporation, Dekalb, IL, USA) were used as sentinel plants for detecting virus spread [16]. Each week, sentinel plants were placed in the field, starting from the second week of April 2020 through the second week of November 2020 for a total of 33 weeks at South and Central AL, and 32 at North AL. In 2021, sentinel plant monitoring began the second week of April and continued through the first week of November for both South and Central AL (31 weeks) and through the end of October for North AL (29 weeks). Plants did not survive cold temperatures outside of this monitoring window. Sentinel plants were maintained in insect-free greenhouses prior to field exposure and were deployed weekly, concurrent with aphid trapping periods (Supplementary Figure S1). After one week, they were removed from the field, sprayed with insecticide, maintained insect-free in the greenhouse for seven to eight weeks, and tested for CLRDV using nested RT-PCR; see Mahas et al. [16] for more details about sentinel plant maintenance and PCR conditions. 

Alate aphids were identified according to methods from Mahas et al. [16] for the eight aphid species: *A. gossypii*, *A. craccivora*, *M. persicae*, *M. euphorbiae*, *P. middletonii*, *R. rufiabdominale*, *S. betae*, and *A. fabae*. Aphids from each trap were removed and counted. The estimated total number of each aphid species per pan trap sample was based on subsamples of 25 aphids. If there were equal to or fewer than 25 aphids per trap, then all alatae were identified. If samples had greater than 25 aphids, then a random subsample of 25 aphids was selected for identification. To randomly select aphids, a 150 mm cell culture dish with 20 mm molded grid (Falcon^®^, Corning, Inc., Corning, NY, USA) was used, with grid sections labeled with a unique number and letter combination. Grids were then selected from a randomly generated list until 25 aphids were collected. The estimated total number of each identified species was calculated by multiplying the proportion identified in the subsample of 25 by the total number of aphids counted.

## 3. Results

### 3.1. The Aphids

Aphids were trapped for a total of 105 continuous weeks; no data were available for the week of 17 September 2020 at South AL due to inclement weather from Hurricane Sally, and no data were available for the week of 20 December 2020 at any region due to the unavailability of collection help over the holidays. A total of 147,656 alate aphids were captured, with 52,164 being a target species (ca. 35.33% of the total). Of the species, *P. middletonii* was by far the most abundant, with an estimated total of 43,351 individuals, 83% of the target aphids. This was followed by *A. gossypii*, with 5403 aphids (10.36%), *R. rufiabdominale*, with 1708 aphids (3.28%), and *M. euphorbiae*, with 824 (1.58%). The remaining species *M. persicae* (506 aphids), *A. craccivora* (361 aphids), *S. betae* (10 aphids), and *A. fabae* (1 aphid) comprised less than 1% each. 

#### 3.1.1. *Aphis gossypii*

**Figure 1:**
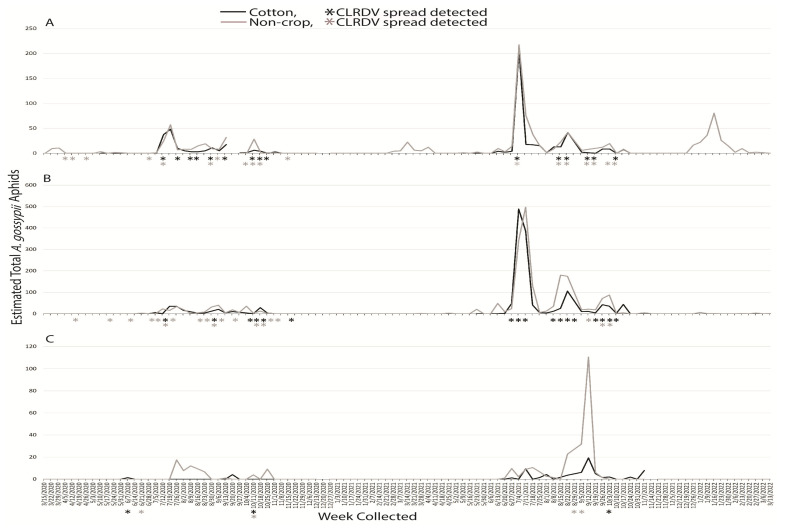
Weekly estimated total number of *Aphis gossypii* identified from traps placed around non-crop (weeds/pasture) and cotton fields at (**A**) Brewton (South AL), (**B**) Shorter (Central AL), and (**C**), Belle Mina (North AL) during two years of trapping. Weekly CLRDV spread was monitored using sentinel plants and identified using RT-PCR beginning the second week of April and continuing through the second and first weeks of November in 2020 and 2021, respectively.

An estimated 5403 *A. gossypii* alatae were captured during the two years: 942 in 2020 and 4461 in 2021. In years one and two, 415 and 1169 were captured at South AL, 454 and 3009 were captured at Central AL, and 73 and 283 alatae were captured at North AL. In the first year, *A. gossypii* alatae were captured for 24 weeks at South AL, 18 weeks at Central AL, and 10 weeks at North AL. The following year, totals were 35, 29, and 18 weeks, at South, Central, and North AL. The week with the highest number captured in any region occurred during 2021 in the week of 11 July with 883 total at Central AL. *Aphis gossypii* had noticeable dispersal events in both years and at all locations when cotton was actively growing (Figure 1). Alatae were intermittently captured in March of both 2020 and 2021 and in January of 2021 at South AL, when they were not identified at the other locations (Figure 1A). *Aphis gossypii* was noticeably absent from traps in North AL from October of 2020 until June of 2021 (Figure 1C). More aphids were captured in the non-crop traps than the crop traps at North AL (Figure 1C).

#### 3.1.2. *Protaphis middletonii*

**Figure 2:**
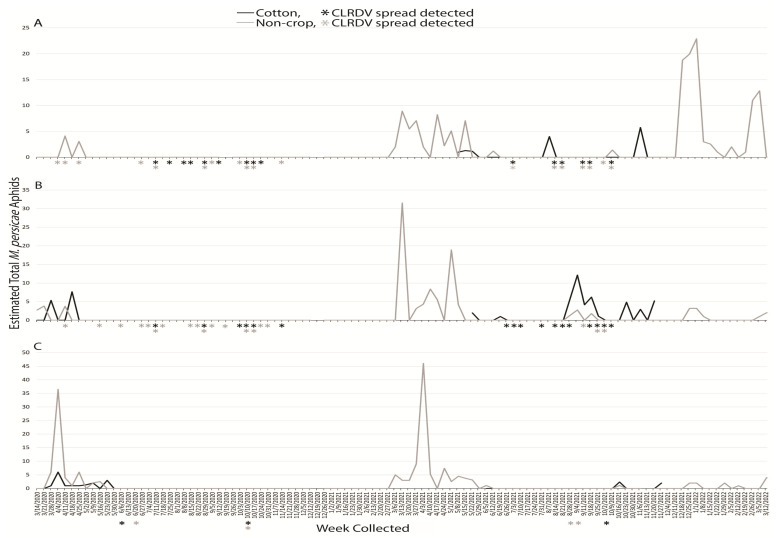
Weekly estimated total number of *P. middletonii* identified from traps placed around non-crop (weeds/pasture) and cotton fields at (**A**) Brewton (South AL), (**B**) Shorter (Central AL), and (**C**), Belle Mina (North AL) during two years of trapping. Weekly CLRDV spread was monitored using sentinel plants and identified using RT-PCR beginning the second week of April and continuing through the second and first weeks of November in 2020 and 2021, respectively.

An estimated total 43,351 *P. middletonii* alatae were captured during the two years: 25,789 and 17,562 for the first and second years, respectively. Broken down correspondingly, 9258 and 5790 were captured at South AL, 11,241 and 8767 were captured at Central AL, and 5444 and 2851 alatae were captured at North AL, respectively. In the first year, *P. middletonii* alatae were captured for 50 weeks at South AL, 46 weeks at Central AL, and 36 weeks at North AL. The following year, these totals were 52, 47, and 40 weeks, respectively, at South, Central, and North AL. The highest numbers of dispersing *P. middletonii* were observed in 2020 and the highest number occurred in a collection period during the week of 4 April at South AL, with a total of 2424 *P. middletonii* captured (Figure 2A,B). Generally, this species was captured in higher numbers from March through May at South and Central AL, and in lower numbers throughout the summer and fall. At North AL, high trap captures were delayed compared to the other locations and occurred from May through September (Figure 2C). The highest number captured at North AL in 2020 was during the week of 1 August (804 alatae), and individuals continued to be captured intermittently through December. Both non-crop and crop habitats tended to capture consistent totals of *P. middletonii* in all regions and years except for 2021 at Central AL, when the majority were dispersing in the non-crop habitat in July and August.

#### 3.1.3. *Rhopalosiphum rufiabdominale*

**Figure 3:**
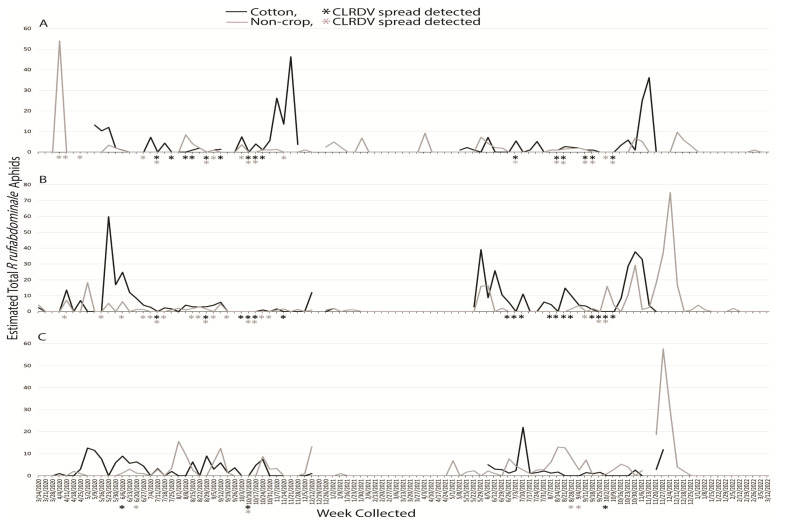
Weekly estimated total number of *R. rufiabdominale* identified from traps placed around non-crop (weeds/pasture) and cotton fields at (**A**) Brewton (South AL), (**B**) Shorter (Central AL), and (**C**), Belle Mina (North AL) during two years of trapping. Weekly CLRDV spread was monitored using sentinel plants and identified using RT-PCR beginning the second and first weeks of November in 2020 and 2021, respectively.

An estimated total 1708 *R. rufiabdominale* alatae were captured during the two years: 735 and 973 for the first and second years, respectively. Broken down correspondingly, 263 and 180 were captured at South AL, 264 and 519 were captured at Central AL, and 208 and 274 alatae were captured at North AL, respectively. In the first year, *R. rufiabdominale* alatae were captured for 27 weeks at South AL, 29 weeks at Central AL, and 28 weeks at North AL. The following year, these totals were 27, 29, and 31 weeks, respectively, at South, Central, and North AL. The number of *R. rufiabdominale* captured any given week ranged from zero to 75, captures occurred from March through December, and numbers of individuals captured were not consistent among years or locations and usually occurred with less than 10 total individuals in any given week (Figure 3). Most were captured in the cotton habitat at South and Central AL and in the non-crop habitat in North AL.

#### 3.1.4. *Aphis craccivora*

**Figure 4:**
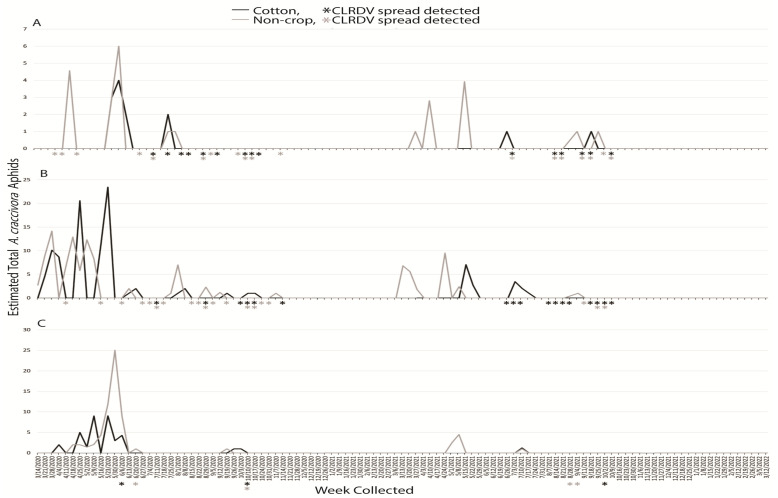
Weekly estimated total number of *A. craccivora* identified from traps placed around non-crop (weeds/pasture) and cotton fields at (**A**) Brewton (South AL), (**B**) Shorter (Central AL), and (**C**), Belle Mina (North AL) during two years of trapping. Weekly CLRDV spread was monitored using sentinel plants and identified using RT-PCR beginning the second week of April and continuing through the second and first weeks of November in 2020 and 2021, respectively.

An estimated total 361 *A. craccivora* alatae were captured during the two years: 297 and 64 for the first and second years, respectively. Broken down correspondingly, 27 and 12 were captured at South AL, 174 and 43 were captured at Central AL, and 96 and 9 alatae were captured at North AL, respectively. In the first year, *A. craccivora* alatae were captured for 6 weeks at South AL, 22 weeks at Central AL, and 13 weeks at North AL. The following year, these totals were 7, 11, and 3 weeks, respectively, at South, Central, and North AL. The highest number captured at any location occurred during the week of 30 May 2020 with 28 aphids at North AL (Figure 4C). No more than 25 total individuals were observed in cotton or non-crop habitats at South and North AL, and this total was no more than 6 individuals at central AL (Figure 4). Aphids captured between non-crop and crop habitat were generally similar. *Aphis craccivora* were typically observed between March and November at all three locations, but the numbers collected were generally low and erratic.

#### 3.1.5. *Macrosiphum euphorbiae*

**Figure 5:**
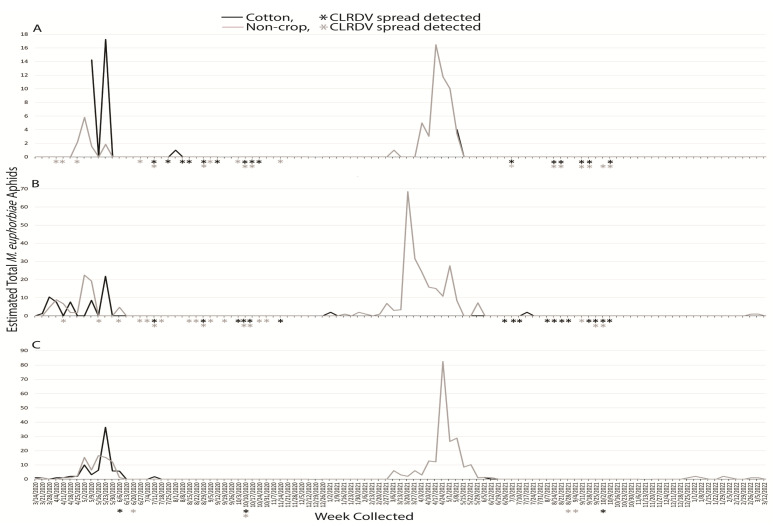
Weekly estimated total number of *M. euphorbiae* identified from traps placed around non-crop (weeds/pasture) and cotton fields at (**A**) Brewton (South AL), (**B**) Shorter (Central AL), and (**C**), Belle Mina (North AL) during two years of trapping. Weekly CLRDV spread was monitored using sentinel plants and identified using RT-PCR beginning the second week of April and continuing through the second and first weeks of November in 2020 and 2021, respectively.

An estimated total 824 of *M. euphorbiae* alatae were captured during the two years: 345 and 479 for the first and second years, respectively. Broken down correspondingly, 45 and 54 were captured at South AL, 145 and 216 were captured at Central AL, and 155 and 209 alatae were captured at North AL, respectively. In the first year, *M. euphorbiae* alatae were captured for 6 weeks at South AL, 17 weeks at Central AL, and 14 weeks at North AL. The following year, these totals were 6, 13, and 21 weeks, respectively, at South, Central, and North AL. The highest number captured in any region occurred during the week of 20 March 2021 with 68 total at Central AL. Activity across the three locations in Alabama predominantly occurred March through May of both years, with few individuals captured during other weeks of the study (Figure 5). While total aphids captured between non-crop and crop habitats varied at South AL, they were consistent in the central and northern parts of the state. In 2021, *M. euphorbiae* alatae were nearly always captured in the non-crop habitat.

#### 3.1.6. *Myzus persicae*

**Figure 6:**
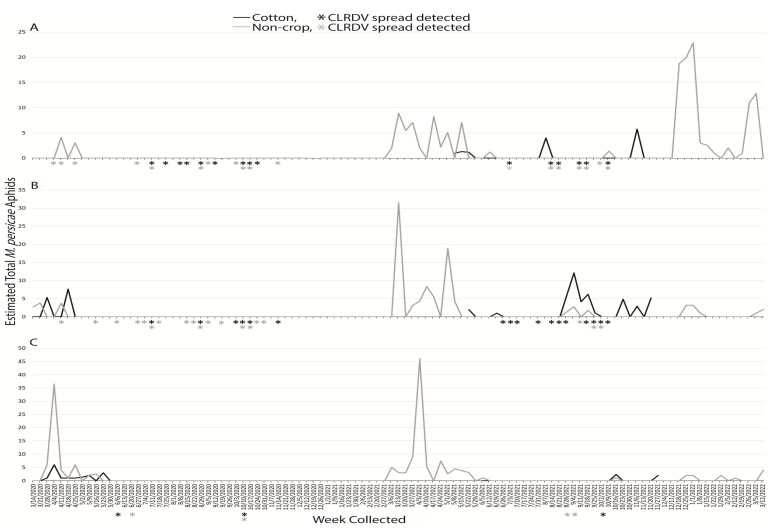
Weekly estimated total number of *M. persicae* identified from traps placed around non-crop (weeds/pasture) and cotton fields at (**A**) Brewton (South AL), (**B**) Shorter (Central AL), and (**C**), Belle Mina (North AL) during two years of trapping. Weekly CLRDV spread was monitored using sentinel plants and identified using RT-PCR beginning the second week of April and continuing through the second and first weeks of November in 2020 and 2021, respectively.

An estimated total of 506 *M. persicae* alatae were captured during the two years: 112 and 394 in the first and second years, respectively. Broken down correspondingly, 9 and 157 were captured at South AL, 23 and 132 were captured at Central AL, and 80 and 105 alatae were captured at North AL, respectively. In the first year, *M. persicae* alatae were captured for 3 weeks at South AL, 5 weeks at Central AL, and 10 weeks at Northern AL. The following year, these totals were 24, 22, and 18 weeks, respectively, at South, Central, and North AL. The highest number of aphids captured in any region occurred the week of 3 April 2021, with 46 total at North AL (Figure 6C). In both years, this species was captured from March through to April or May at all locations. In 2020, *M. persicae* was not captured after that time, but from 2021, intermittent captures occurred throughout the remainder of the study, with the exception of July 2021 when no captures occurred at any location (Figure 6). Alatae were captured in non-crop habitats early in the year (March–April) before cotton was planted but appeared in crop habitats in August and November.

#### 3.1.7. *Aphis fabae* and *Smythurodes betae*

Too few individuals of *A. fabae* and *S. betae* were captured to make any conclusive determination about their population dynamics. A single *S. fabae* alate was captured in February of 2021 at Central AL. Fewer than 10 *S. betae* alatae were collected across both 2020 and 2021; one was collected at South AL during the first week of August 2021, and all others were caught at Central AL from the last week of March through April.

### 3.2. Sentinel Plants

Sentinel plant monitoring was used to identify weeks during the year that CLRDV was spread by aphids to cotton at the three locations. CLRDV spread was detected in more weeks during 2020 than in 2021. Viral spread occurred the first week of April in 2020, which corresponded with the first week that sentinel plants were deployed at South and Central AL, and before cotton was planted. The spread of the virus was detected throughout the growing season and after the growing season until sentinel plant monitoring was terminated in November. At North AL, viral spread was only detected in three weeks during 2020 in June and October. 

Sentinel plants were deployed concurrent with aphid trapping to identify which species were present when virus spread occurred. The reported vector of CLRDV, *A. gossypii*, was captured during weeks when viral spread was detected during the growing seasons, and successive weeks of virus spread typically began coincident with a peak in trap captures of the vector (Figure 1). This was not true for virus spread events observed in April and November of 2020 at South and Central AL (Figure 1). *Protaphis middletonii* was the most abundant species present during the weeks of April and May 2020 (Figure 2). Other species were also present, but not during all weeks (Figure 3, Figure 4, Figure 5 and Figure 6). The week that virus spread was detected in November of 2020 at South and Central AL, *P. middletonii* and *R. rufiabdominale* were the only species captured. The numbers of *P. middletonii* (Figure 2) were 61 at South AL and 15 at Central AL, and the numbers of *R. rufiabdominale* (Figure 3) were 15 at South AL and 0 at Central AL 

## 4. Discussion

This study provides basic information regarding the epidemiology and management of CLRDV by identifying when virus spread occurs in three distinct cotton growing regions, concurrent with characterizing dispersal dynamics of known and potential aphid vectors. This work supports and expands upon previous research by Mahas et al. [16], who demonstrated the presence of six cotton-feeding aphid species at the same south AL site from May through August. All eight cotton-feeding aphid species were collected in this study. In both years, *P. middletonii* was the most abundant, followed by *A. gossypii*. The remaining species were less abundant, and most were not present throughout the cotton growing season (Figure 1, Figure 2, Figure 3, Figure 4, Figure 5 and Figure 6). Sentinel plant monitoring in both cotton and non-crop habitats identified multiple CLRDV spread events and suggests that CLRDV spread is occurring widely in the environment.

These results support *A. gossypii* as the primary vector of CLRDV in these agroecosystems. *Aphis gossypii* is known to colonize cotton annually in Alabama from late June to mid-July. Although populations do not persist and continue to build after this time [24], this study demonstrates that CLRDV spread occurs throughout the summer, concurrent with *A. gossypii* dispersal events (Figure 1). This provides multiple opportunities for virus spread to occur from the landscape to the crop and is one possible explanation for the large end-of-season incidence of CLRDV reported for locations in Alabama and Georgia [11,16]. 

Our *P. middletonii* data shows that it is active at a time in early spring at both South and Central AL sites when CLRDV spread is occurring and *A. gossypii* is not abundant. This species is known to infrequently feed on cotton and is often found on the roots of host plants, tended by ants [31]. The ability of this species to transmit CLRDV is unknown. If proven to transmit the virus, even among host reservoirs, the large number of dispersing aphids in spring suggests this species could serve as a transient vector to cotton through short-term feeding periods. The longer duration of these activity peaks, lasting for weeks to months in this study, could contribute to increased virus incidence in cotton. Future studies are needed to test the vector competence of *P. middletonii*. 

The other six cotton-feeding aphid species represented only 2.31% of all captures. The presence of *A. fabae* (Central AL) and *S. betae* (South and Central AL) was confirmed, but the small number of captures suggests minimal to no contribution to CLRDV spread in this study. *Rhopalosiphum rufiabdominale* was consistently detected in spring but largely absent afterward (Figure 3). This species was present during the weeks of early season virus spread, but its ability to transmit CLRDV is unknown. *Aphis craccivora*, *M. persicae*, and *M. euphorbiae* were not abundant, or observed, during summer months in this study when the majority of CLRDV spread occurred. More alatae were captured as latitude increased (Figure 4, Figure 5 and Figure 6), consistent with their optimal developmental temperatures of 20–25 °C with a sharp decrease at 30 °C [32,33].

A recent study showed that U.S. populations of *A. craccivora* and *M. persicae* did not acquire CLRDV from or transmit CLRDV to cotton [20]. In that study, mature cotton was not an ideal host for *A. craccivora*, and the population of *M. persicae* had been maintained in a laboratory for many years and was not originally collected from the cotton belt. Although the results of [20] support these species not contributing to virus spread to cotton, it is not clear whether vector competent aphid populations could transmit CLRDV among reservoir hosts that better support aphid feeding and reproduction. It is not possible to determine from this study whether either species contributed to observed virus spread, but species with low abundance are not expected to contribute significantly to end-of-season virus incidence [34]. Future studies are needed to test the vector competence of *R. rufiabdominale* and *M. euphorbiae*.

Although management is not the focus of this study, these results can be used to inform the development of strategies to reduce the spread of CLRDV. Currently, there is not a management plan for CLRDV due to a lack of information about its symptomatology, yield, losses, efficacy of different practices, and virus spread. One study showed that managing aphid populations with frequent insecticide sprays did not reduce final CLRDV incidence in south Alabama and south Georgia, where incidence ranged from 60–100% in research plots [16]. One possible explanation is that vector management occurred from late June through mid-July, but CLRDV spread can continue [16]. It is difficult to disrupt CLRDV spread because it occurs so frequently; in both years of this study, viral spread was detected throughout the growing season at South and Central AL sites. This data suggests that the development of resistant varieties is the most effective strategy to prevent infection. Yield losses are not commonly perceived to be associated with CLRDV in infected commercial fields [35,36,37,38], even when incidence is high (Jacobson and Conner, personal observation). This suggests that mature plant resistance and/or good plant health management strategies may reduce the incidence of disease caused by CLRDV and the probability of yield loss outcomes. More research is needed to understand the virus–plant–environment interactions and conditions that lead to yield loss outcomes.

Our study provides the first multi-year data in cotton agroecosystems on the population dynamics of all eight aphid species reported to colonize cotton. A major finding was that CLRDV spread occurs regularly from spring through fall, before, during, and after the cotton growing season in central and south Alabama. During the cotton growing season, *A. gossypii* is the most abundant species, and these data support *A. gossypii* as the primary vector for CLRDV based on the timing of dispersal events in conjunction with weeks when viral spread was detected. The early spread indicates that CLRDV weedy host reservoirs serve as inoculum sources and suggests that additional aphid species are vectors of CLRDV, because *A. gossypii* is not active during these early spring months. *Protaphis middletonii* is of special interest because it was the most abundant aphid identified in early spring, when initial CLRDV spread occurred. The North AL site had the fewest weeks of CLRDV spread, and the numbers of *A. gossypii* captured were lower. The three locations were chosen to represent distinct growing regions that are not unique to Alabama. The ecoregion that encompasses the north AL location spans other cotton producing states, where no to low incidence of CLRDV has been reported [11]. Similarly, South and Central AL locations share ecoregions with other areas of the southeast, including Georgia, where up to 100% CLRDV incidence has been reported [16]. Future studies are needed to characterize the vector competence of additional aphids and to develop durable management strategies for protecting plant health.

## Figures and Tables

**Table 1:** Cotton harvest and planting dates (month/day) for the fields where aphid and sentinel plant monitoring occurred.

	Cotton Plant Date		Cotton Harvest Date	
	2020	2021	2020	2021
South AL (BARU)	5/6, 6/1	5/28	10/14	10/19, 11/14
Central AL (EVS)	5/26	4/30	10/27	11/17–11/19
North AL (TVREC)	5/26	5/27	11/9	11/16

## Data Availability

Data will be provided upon request.

## Supplementary Materials

The following supporting information can be downloaded at: https://www.mdpi.com/article/10.3390/insects14070604/s1, Figure S1: Sentinel plants with 2–3 true leaves ready for deployment into the field (A) and a yellow pan trap set out to collect aphids (B); Table S1: The number of plants that tested positive for CLRDV out of the total tested for that location, week collected, and habitat (crop and non-crop).
