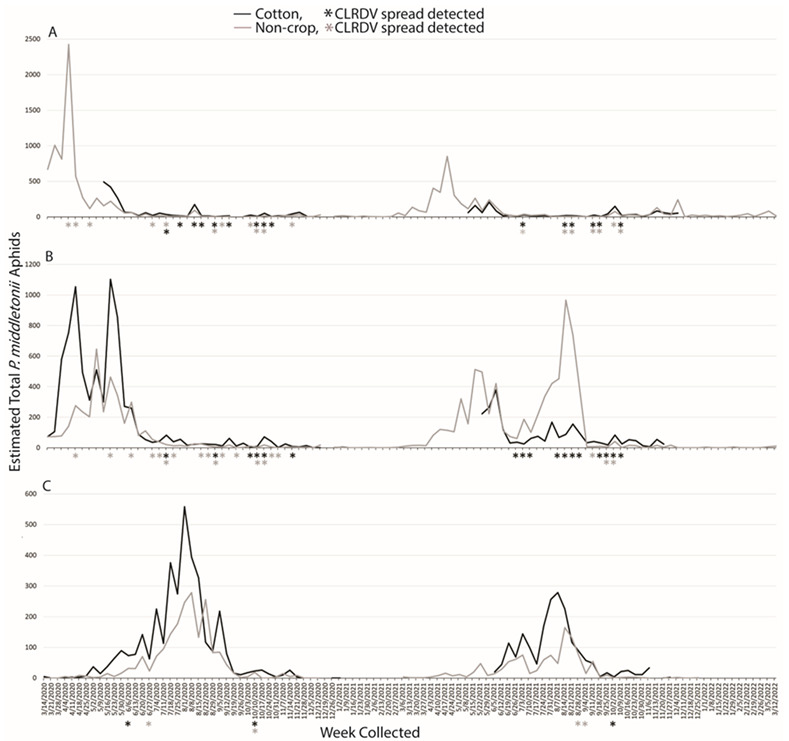# Correction: Mahas et al. Seasonal Dynamics of Aphid Flights and Cotton Leafroll Dwarf Virus Spread in Alabama. *Insects* 2023, *14*, 604

**DOI:** 10.3390/insects14120925

**Published:** 2023-12-05

**Authors:** Jessica B. Mahas, Charles Ray, Adam Kesheimer, Kassie Conner, Alana L. Jacobson

**Affiliations:** 1 Department of Entomology and Plant Pathology, Auburn University, 301 Funchess Hall, Auburn, AL 36849, USA; jba0022@auburn.edu (J.B.M.); chr138@msstate.edu (C.R.); ajk0055@auburn.edu (A.K.); 2 Alabama Cooperative Extension, 961 S. Donahue Dr., Auburn, AL 36849, USA; connekn@auburn.edu

## Error in Figure

In the original publication [1], there was a mistake in Figure 2 as published. The graph for Figure 6 was uploaded in its place instead of the correct graph. The corrected Figure 2 appears below. The authors state that the scientific conclusions are unaffected. This correction was approved by the Academic Editor. The original publication has also been updated.