# Supplementary material for: Seasonal Dynamics of Aphid Flights and Cotton Leafroll Dwarf Virus Spread in Alabama
Source: Insects. 2023 Jul 4;14(7):604. doi: 10.3390/insects14070604 (PMC10380835; doi:10.3390/insects14070604)
Supplement: Supplementary file 1 [file insects-14-00604-s001.zip › insects-2450094-supplementary.pdf]

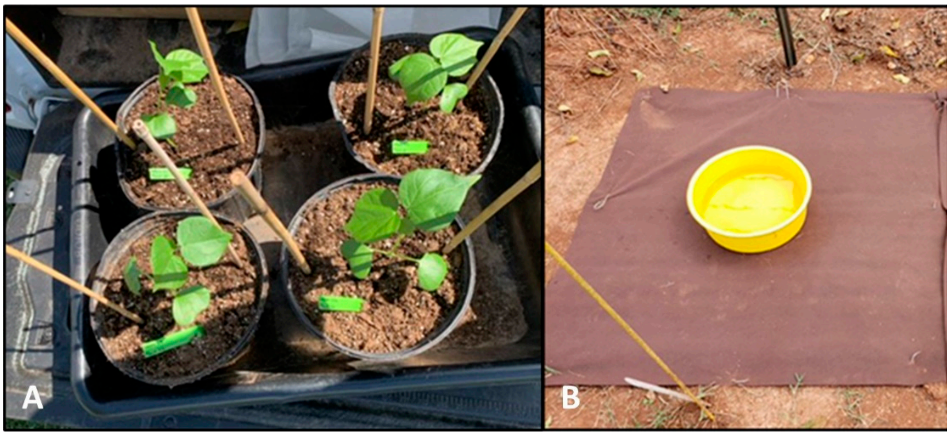

Figure S1. Sentinel plants with 2-3 true leaves ready for deployment into the field (A) and a yellow pan trap set out to collect aphids (B).

Table S1. The number of plants that tested positive for CLRDV out of the total tested for that location, week collected, and habitat (crop and non-crop).

	2020			2021		
Location	Week of monitoring	Cotton	Non-crop	Week of monitoring	Cotton	Non-crop
South AL (BARU)	4/6 - 4/12	0/0	4*/4	4/12 - 4/18	0/0	0/4
Central AL (EVS)		0/4	0/4		0/0	0/4
North AL (TVREC)		0/4	0/4		0/0	0/4
South AL (BARU)	4/13 - 4/19	0/0	4*/4	4/19 - 4/25	0/0	0/4
Central AL (EVS)		0/4	2*/2		0/0	0/4
North AL (TVREC)		0/0	0/0		0/0	0/1
South AL (BARU)	4/20 - 4/26	0/0	0/4	4/26 - 5/2	0/0	0/4
Central AL (EVS)		0/0	0/4		0/0	0/3
North AL (TVREC)		0/2	0/0		0/0	0/4
South AL (BARU)	4/27 - 5/3	0/0	4*/4	5/3 - 5/9	0/0	0/4
Central AL (EVS)		0/4	0/4		0/0	0/3
North AL (TVREC)		0/4	0/4		0/0	0/4
South AL (BARU)	5/4 - 5/10	0/0	0/4	5/10 - 5/16	0/4	0/3
Central AL (EVS)		0/4	0/4		0/0	0/2
North AL (TVREC)		0/1	0/2		0/0	0/3
South AL (BARU)	5/11 - 5/ 16	0/2	0/4	5/17 - 5/22	0/4	0/4
Central AL (EVS)		0/3	0/4		0/0	0/4
North AL (TVREC)		0/0	0/0		0/0	0/4
South AL (BARU)	5/17 - 5/23	0/2	0/4	5/23 - 5/29	0/4	0/4
Central AL (EVS)		0/3	4*/4		0/0	0/4
North AL (TVREC)		0/4	0/4		0/0	0/4
South AL (BARU)	5/24 - 5/29	0/3	0/3	5/30 - 6/5	0/4	0/4
Central AL (EVS)		0/4	0/4		0/4	0/3

North AL (TVREC)		0/4	0/4		0/0	0/4
South AL (BARU)	5/30 - 6/6	0/4	0/4	6/6 - 6/12	0/4	0/4
Central AL (EVS)		0/2	0/4		0/4	0/4
North AL (TVREC)		0/4	0/3		0/4	0/4
South AL (BARU)	6/7 - 6/13	0/3	0/4	6/13 - 6/19	0/4	0/4
Central AL (EVS)		0/3	3*/3		0/4	0/4
North AL (TVREC)		3*/3	0/4		0/4	0/4
South AL (BARU)	6/14 - 6/20	0/3	0/4	6/20 - 6/26	0/4	0/4
Central AL (EVS)		0/4	0/4		0/4	0/4
North AL (TVREC)		0/3	0/4		0/4	0/4
South AL (BARU)	6/21 - 6/27	0/4	0/4	6/27 - 7/4	0/4	0/4
Central AL (EVS)		0/4	0/4		1/4	0/4
North AL (TVREC)		0/4	4*/4		0/4	0/4
South AL (BARU)	6/28 - 7/4	0/4	4*/4	7/5 - 7/10	2/4	1/4
Central AL (EVS)		0/4	4*/4		3/4	0/4
North AL (TVREC)		0/4	0/4		0/4	0/4
South AL (BARU)	7/5 - 7/11	0/4	0/4	7/11 - 7/17	0/4	0/4
Central AL (EVS)		0/4	1/4		2/4	0/4
North AL (TVREC)		0/4	0/4		0/4	0/4
South AL (BARU)	7/12 - 7/18	2/4	2/4	7/18 - 7/24	0/4	0/4
Central AL (EVS)		2/4	1/4		0/4	0/4
North AL (TVREC)		0/4	0/4		0/4	0/3
South AL (BARU)	7/19 - 7/25	0/2	0/4	7/25 - 7/30	0/4	0/4
Central AL (EVS)		0/4	1/4		0/4	0/3
North AL (TVREC)		0/4	0/3		0/4	0/4
South AL (BARU)	7/26 - 8/1	1/4	0/4	8/1 - 8/7	0/4	0/4
Central AL (EVS)		0/4	0/4		0/4	0/3
North AL (TVREC)		0/4	0/4		0/4	0/4
South AL (BARU)	8/2 - 8/8	0/3	0/4	8/8 - 8/14	0/4	0/3
Central AL (EVS)		0/4	0/4		3/4	0/3
North AL (TVREC)		0/4	0/4		0/3	0/4
South AL (BARU)	8/9 - 8/15	4*/4	0/4	8/15 - 8/21	1/4	1/3
Central AL (EVS)		0/4	0/4		4/4	0/4
North AL (TVREC)		0/3	0/4		0/4	0/4
South AL (BARU)	8/16 - 8/22	1/4	0/4	8/22 - 8/28	2/4	1/4
Central AL (EVS)		0/4	1/4		2/4	1/4
North AL (TVREC)		0/3	0/4		0/4	0/4
South AL (BARU)	8/23 - 8/29	0/4	1/4	8/29 - 9/4	0/4	0/4
Central AL (EVS)		0/4	0/4		1/4	0/4
North AL (TVREC)		0/4	0/4		0/4	1/4
South AL (BARU)	8/30 - 9/5	2/4	1/4	9/5 - 9/11	0/4	0/4

Central AL (EVS)		2/3	1/3		0/4	0/2
North AL (TVREC)		0/4	0/4		0/4	1/4
South AL (BARU)	9/6 - 9/12	0/4	1/4	9/12 - 9/18	2/4	1/4
Central AL (EVS)		0/4	1/4		0/4	1/4
North AL (TVREC)		0/4	0/4		0/4	0/4
South AL (BARU)	9/13 - 9/19	1/3	0/4	9/19 - 9/25	1/3	3/4
Central AL (EVS)		0/3	0/4		2/4	0/4
North AL (TVREC)		0/4	0/4		0/4	0/4
South AL (BARU)	9/20 - 9/25	0/3	0/4	9/26 - 10/2	0/2	0/4
Central AL (EVS)		0/4	1/4		3/3	1/4
North AL (TVREC)		0/4	0/4		0/0	0/0
South AL (BARU)	9/26 - 10/3	0/0	0/0	10/3 - 10/9	0/4	1/4
Central AL (EVS)		0/4	0/2		4/4	3/4
North AL (TVREC)		0/4	0/3		2/4	0/4
South AL (BARU)	10/4 - 10/10	0/2	3/4	10/10 - 10/16	1/4	1/4
Central AL (EVS)		2/4	0/4		3/4	0/4
North AL (TVREC)		0/3	0/1		0/1	0/4
South AL (BARU)	10/11 - 10/17	4/4	4/4	10/17 - 10/23	0/3	0/4
Central AL (EVS)		2/4	1/4		0/0	0/4
North AL (TVREC)		2/4	1/4		0/4	0/2
South AL (BARU)	10/18 - 10/24	2/4	2/4	10/24 - 10/30	0/1	0/4
Central AL (EVS)		2/4	1/4		0/0	0/4
North AL (TVREC)		0/1	0/0		0/3	0/4
South AL (BARU)	10/25 - 10/31	1/2	0/4	10/31 - 11/6	0/4	0/4
Central AL (EVS)		0/4	1/4		0/4	0/3
North AL (TVREC)		0/4	0/4		0/0	0/0
South AL (BARU)	11/1 - 11/7	0/1	0/3	11/7 - 11/13	0/2	0/1
Central AL (EVS)		0/4	1/4		0/1	0/0
North AL (TVREC)		0/0	0/0		0/0	0/0
South AL (BARU)	11/8 - 11/14	0/1	0/0	11/14 - 11/20	0/0	0/0
Central AL (EVS)		0/0	0/0		0/0	0/0
North AL (TVREC)		0/2	0/3		0/0	0/0
South AL (BARU)	11/15 - 11/21	0/4	0/4	11/21 - 11/27	0/0	0/0
Central AL (EVS)		4*/4	0/4		0/0	0/0
North AL (TVREC)		0/0	0/0		0/0	0/0
South AL (BARU)	11/22 - 11/28	0/1	0/0			
Central AL (EVS)		0/0	0/0			
North AL (TVREC)		0/0	0/0			

*Indicates at least one plant tested positive in cohort testing, but individual plants were not able to be tested.
